# Importance of local knowledge in plant resources management and conservation in two protected areas from Trás-os-Montes, Portugal

**DOI:** 10.1186/1746-4269-7-36

**Published:** 2011-11-23

**Authors:** Ana Maria Carvalho, Amélia Frazão-Moreira

**Affiliations:** 1 CIMO (Centro de Investigação de Montanha), Dept. Biologia e Biotecnologia, Escola Superior Agrária, Instituto Politécnico de Bragança, 5301-855 Bragança, Portugal; 2 CRIA (Centro em Rede de Investigação em Antropologia), Dept. de Antropologia, Faculdade Ciências Sociais e Humanas da Universidade Nova de Lisboa, Av. de Berna, 26 C, 1069-061 Lisboa, Portugal

**Keywords:** Local knowledge, cultural heritage, protected areas, Montesinho, Douro International, Portugal

## Abstract

Many European protected areas were legally created to preserve and maintain biological diversity, unique natural features and associated cultural heritage. Built over centuries as a result of geographical and historical factors interacting with human activity, these territories are reservoirs of resources, practices and knowledge that have been the essential basis of their creation. Under social and economical transformations several components of such areas tend to be affected and their protection status endangered.

Carrying out ethnobotanical surveys and extensive field work using anthropological methodologies, particularly with key-informants, we report changes observed and perceived in two natural parks in Trás-os-Montes, Portugal, that affect local plant-use systems and consequently local knowledge. By means of informants' testimonies and of our own observation and experience we discuss the importance of local knowledge and of local communities' participation to protected areas design, management and maintenance. We confirm that local knowledge provides new insights and opportunities for sustainable and multipurpose use of resources and offers contemporary strategies for preserving cultural and ecological diversity, which are the main purposes and challenges of protected areas. To be successful it is absolutely necessary to make people active participants, not simply integrate and validate their knowledge and expertise. Local knowledge is also an interesting tool for educational and promotional programs.

## Background

Local knowledge (LK) has great cultural significance and refers to the use of many wild or domesticated resources and the management of natural habitats and agroecosystems. LK refers, as well, to some other important rural activities and practices, such as cattle transhumance, agricultural techniques (e.g. crop rotation, irrigation methods, multi use parcels and partial harvest), land management (e.g. ownership, land holding fragmentation, terraces, natural or artificial boundaries), rituals and ceremonies, oral traditions and symbolism, communitarian features and settlement patterns [1-5]. However, LK is not static; LK is dynamic and evolves exploring the full potential of human-environmental interactions, experimenting and learning from others and adapting to change over time [6].

Several authors [7-11] emphasize the advantages of including careful studies on local patterns of plant use in the conceptual issues involved in the management of protected areas and in conservation biology, although some researchers and managers enrolled have experienced serious difficulties to incorporate them into conservation strategies [10,12].

The importance of natural environment and of cultural landscapes and heritage has increased in the last years. National and European authorities for nature conservation have been engaged in comprehensive resource networks and effective legislation and regulations for protected areas [13,14] putting together different efforts to sustain biodiversity and to enlist the full range of partners implicated in such a process. It appears that the involvement and participation of local communities was the essential basis on which protected areas would build a system of management which has integrity, security and success, particularly those including human settlements [15]. Nevertheless, conservation measures were mostly designed by outsiders who were culturally detached and parks boundaries were mainly based on environmental criteria [12,16].

**Figure 1:**
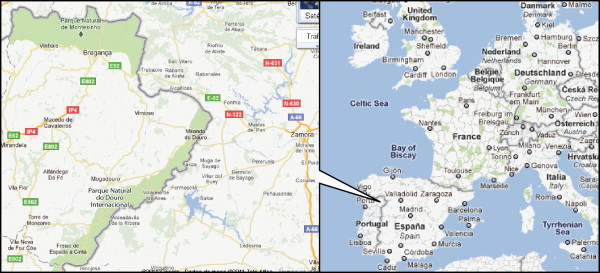
**Studied sites (green shaded) location map: Parque Natural de Montesinho (Vinhais and Bragança) and Parque Natural Douro Internacional (Miranda do Douro), Trás-os-Montes, Portugal**. Adapted from Google maps.

Two important natural protected areas of Portugal (Figure 1), located in the most northeastern part of the country (Trás-os-Montes), have a great diversity of natural and semi-natural habitats (e.g. deciduous woodlands and firebreaks or grasslands managed as grazing or cutting) and humanized landscapes which are repositories of natural life and cultural heritage. The territories of the Natural Park of Montesinho (PNM) and the Natural Park of Douro International (PNDI) are the result of many geographical and historical factors and represent harmonious integration of human activity with nature, allowing ecological diversity to be maintained and valued. A very long history of human occupation and management of natural resources, as well as economic circumstances leading to predominantly rural structure until recently, built up a rich local knowledge that shaped landscape and enabled certain species and habitats to remain relatively stable.

Nowadays, Portuguese rural contexts, including those of these protected areas (PNM and PNDI) experience sudden and faster social and economical transformations and rural landscapes are rapidly changing and diversifying, accordingly. These changes have promoted a loss of cultural heritage and are affecting both LK and the systems of plant-use [16,17], as well as traditional landscapes [18], agro ecosystems biodiversity and the flora and fauna habitats for whose conservation the parks were created. Factors involving landscape/habitat changes will have serious long-term repercussions and may influence the status of such protected areas.

Based on different inquiry techniques conducted for ten years and on key-informants' opinions we explore how natural parks landscapes reflect LK and why LK is important to natural resources management and conservation, above all to those resources concerning food and medicinal plants. Moreover we discuss landscape changes locally observed and perceived in Trás-os-Montes, Portugal, which directly concern LK and we report how useful LK can be to improve educational strategies and policies, through the example of the creation of the Ecomuseum of Terra de Miranda (Picote, Trás-os-Montes, Portugal).

## Methods

The studied area (Figure 1) is mostly mountainous with small villages (many of them less than 100 inhabitants) scattered all over the landscape and corresponding to Vinhais, Bragança and Miranda do Douro municipalities. The Montesinho Natural Park was set up in 1979. It stretches across almost 750 km^2 ^along the Montesinho and Coroa hills on the border with Spain and contains 91 villages with altogether 8,000 inhabitants [19]. The Douro International Natural Park was set up in 1998. It covers an area of about 860 km^2^, with 35 villages and nearly 14,300 inhabitants, along the riverbanks of Douro and Águeda to the south (about 120 km long) [20].

The local economy was based on forestry (important tree resources are oaks, chestnuts, wild cherries, walnuts), pastoralism (cattle, sheep and goats), small-scale animal breeding (pigs and poultry), fishing and hunting and on small farming systems, with important crop production diversity (e.g. grains, potatoes, cabbages, beans, pumpkins and squashes, fodder), and a high level of subsistence strategies avoiding productive risks (e.g. community plots, multipurpose parcels, crop rotation, mixed crops, landraces and farmer varieties). Natural surroundings were explored and provided staple products often (e.g. medicinal and edible plants, mushrooms, berries) used as food and to prepare homemade remedies for human primary healthcare and animal diseases. Besides food and fodder, arable crops, scrubland and woods supplied other basic needs, such as fuel, domestic tools, textiles and building raw materials. At times, surpluses of grains, chestnuts, potatoes, livestock, textiles, handicrafts, charcoal and wood were traded or sold, to generate extra income. Mining, smuggling and other men activities complemented the household income [16-18,21].

Affected by agriculture abandonment and both population ageing and erosion, due to two decades of disincentives measures and distortions created by the Common Agricultural Policy and several migratory flows, many small villages, particularly in Bragança municipality, have become devitalized and dormitories of the nearest towns (e.g. Bragança and Miranda do Douro), their inhabitants had to find different jobs and very few are still full-time farmers. Households hardly depend on farming incomes. Many have become absentee owners and their lands have been afforested [16,18,21,22].

Several ethnobotanical surveys were undertaken for almost ten years (2000-2010) in the two natural parks territories (PNM and PNDI) within the scope of three research projects that aimed to record and document traditional knowledge on plant-use, related technologies and management of natural resources [21-23]. Different techniques of inquiry, such as semi-structured and structured interviews, participant observation, group discussion, free-listing and pile-sorting, were used to collect data [3,24,25]. Considering those communities that had a history of agropastoral activities and homegardens until very recently (2005), a stratified sampling of approximately 40% of the villages in the study area was used. In every case study, at least informal interviews [25], consented semi-structured interviews and participant observation were conducted during all seasons of the year. Informants (a total of 185, 65% women, mean age 65, maximum 93 and minimum 10 years old) were selected using random sample and snow-ball methods [3,24]. For particular purposes, in-depth interviews have been held with 30 local experts or key-informants (informants with profound knowledge of a particular aspect of local culture, e.g. shepherds, smugglers, hunters, healers). This sub-sample was intentionally selected from those informants considered knowledgeable by their neighbors.

The key-informants sample included 18 women and 12 men, nearly all over 60 years old (mean age 67), having lived most of their lives in the selected villages, being acquainted with forestry, animal husbandry, agricultural practices and local farming systems and culture, and that were able to remember or have participated in different management scenarios of natural and traditional landscape (plows, clearings, common lands, forestation, road system, irrigation canals and mining, for instance).

The information for this paper topic is based on key-informants' discourses carried out along a ten year period, refined with the authors' observations and reflections. Contacted in different occasions, at home, in the gardens and arable fields, during tours on the villages surroundings or while herding, using informal talks and semi-structured interviews (some of them recorded with informants permission), key-informants were invited to speak about the villages lifestyles and livelihood over the last thirty years and, subsequently, before and after the parks creation. They shared their experiences and views and commented extensively particular issues, such as LK on plant-use, emblematic wild species and crop varieties, traditional versus modern cropping practices, land-use patterns, farming systems, resources management, landscapes changes, agricultural policies, infrastructures and facilities, and demographic trends. Moreover, they were encouraged to tell about their personal experience with the parks authorities and to express their opinion about the management of these protected areas.

All the interviews and interactions were performed in Portuguese although in the small villages of Miranda do Douro, the Mirandese language (belonging to the Astur-Leonese linguistic group) is also spoken by many inhabitants.

We set out an ethnobotanical database to organize and record the information and we created a photographic collection which we used to compare structural components of traditional landscapes and some elements such as land cover, building and infrastructural construction for a time period of almost a decade.

## Results

A descriptive and qualitative analysis of the reported data shows that all the key-informants were well aware of the importance of LK which they view as an essential tool to maintain cultural contexts and natural resources to use in a traditional way or to incorporate in innovative alternatives, such as new produces or agrotourism. Moreover, they sustained that landscape changing in structure and land use has been locally detected in the last two decades in all the studied area, and that some of the causes are related to different trends in agriculture (e.g. mechanization) and ways of living (e.g. compulsory education for children, better mobility and new concept of residential housing). Some highlighted a general lack of awareness about LK as a possibility (i.e. people does not know how to do things!) and also mentioned the influence and the status of modern global lifestyles (i.e. LK often been linked to past and hard times); a few pointed out the applicability of LK in a current context (i.e. some uses and practices are viewed as obsolete).

Key-informants expressed concern about LK persistence because most of them think that some resources conservation is highly dependent on continuous use and practices. For instance, they confirmed that edible, medicinal and aromatic plants wild gathering is supported by extensive knowledge on species differentiation and identification and on particular criteria of use, that guarantee quality and safety for consumers. Abandonment of some practices and crops affects certain species that depend on them. In the absence of grazing and clearing, flora composition and useful species availability is rather different in meadows and woodlands. Table 1 describes some examples that illustrate how key- informants explained the close relationship between LK (knowledge and practices), the use and the conservation of edibles and medicinal plants. According to them this link is also important to mushrooms gather and consumption.

**Table 1:** Why common uses and conservation of certain edible and medicinal plants depend on local knowledge? Examples consider the best quality materials and safety for users.

Species Latin name Species common name (PT/GB)^1^	Main use	Useful LK	Practices that promote conservation & best use^2^
*Arnica montana *L. arnica/mountain arnica *Fragaria vesca *L. amiródio/wild strawberry *Physospermum cornubiense *(L.) DC. anis/bladderseed *Tuberaria lignosa *(Sweet) Samp. alcária/similar to rockrose	Medicinal Claimed to have potent anti-inflammatory effect and high demanded	Grown in very specifics habitats. Inconspicuous, are unnoticed. Skillful gathers find them searching some particular soil characteristics. Few people know the best sites, gathering periods, plant grow stages and how to prepare remedies	Maintaining a balance between wilderness and extensive management of woodlands and scrublands, promoting some disturbance to limit competition but avoiding great disturbances, resulting in the removal of large amounts of biomass

*Borago officinalis *L. borragem/borage *Bryonia dioica *Jacq. norça/white bryony *Crataegus monogyna *Jacq. espinheiro/hawthorn *Origanum vulgare *L. *oregão/oregano*	Food and medicinal Supplements, restoratives and condiments Digestive, respiratory, cardiovascular affections	Different parts and different grow stages of the same plant may be used for several purposes and prepared in many ways, according to specific circumstances and need s.	Extensive farming, avoiding pesticides and fertilizers Clearing, mowing, maintaining pathways, road slopes and properties borders (vegetation and traditional stone walls)
			
*Montia fontana *L. merujes/water blinks *Portulaca oleracea *L beldroega/green purslane	Food Consumed mainly raw	Abilities to clearly identify morphological features and convenient gathering sites are fundamental	Surface water management Cleaning of pathways Extensive farming

*Arenaria montana *L. seixinha/mountain sandwor *Thymus pulegioides *L. pojinha/broad-leaved thym *Chondrilla juncea *L. ginjeira/nakedweed	Medicinal Medicinal Food and medicinal	The best material should be gathered in managed sites such as clearings in oak forests, crop lands and meadows, which involve knowledge and proficiency	Woodlots and meadows management and extensive farming, avoiding pesticides and fertilizers

*Geranium robertianum *L. Erva- de- S. Roberto/herb Robert *Hypericum perforatum *L. piricão fêmea/St John's wort	Medicinal Oral use Digestive system and diuretic	Skills to identify certain morphological characters and to avoid confusion among similar species, with no medicinal value	Keeping traditional land tenure (communal versus fenced individual plots) and natural boundaries, preserving the best habitat and material

*Tamus communis *L. black bryonie	Food and medicinal	Toxic plants. Both gathering and uses require expertise to guarantee consumers safety	Woodland management as fruiting plants (used for remedies) are only available in clearings
			
*Pterospartum tridentatum *(L.) Willk carqueja/similar to brooms			Scrubland management avoiding fires and invasive species

1. For each species we present the most cited common name although there are others in the studied area.

2. The presence of some of these species is really affected by abandonment and changing in cover and agricultural practices. Nevertheless, according to our informants, those still occurring and existing, have different food and medicinal properties which may have impact in quality and safety. LK is needed to an integrated and sustainable management of habitats and plant materials.

They also focused upon changing processes imposed by environmental and agricultural policies, by rules and regulations in protected areas and by socioeconomic measures that affected their lives, their homestead, had impact on LK persistence and transmission and caused landscape modifications.

None of the key-informants did ever have any conflict with the parks authorities but although they have not clearly commented the parks policies, they disagreed with measures restricting several activities, such as hunting or fishing, wild gathering and forest management which they see as an economic constrain, a disincentive to collaborate on conservation purposes and an imposition that should not be applied to residents.

Main signs of landscape changes identified by key-informants are new infrastructures and facilities, demographic trends and different land use and cover which were mentioned as follows:

(i) The emergence of a road network since the 1990s allowing a better physical communication between some villages and nearest towns, as it was very inefficient or nonexistent in many of cases. Obviously, all key-informants referred to the road system as a very important benefit but also, as the greatest change due to land expropriation and fragmentation and to visual impact (i.e. roads overlap in landscape);

(ii) The village planning since 1975. For instance, household sewage and urban waste water, water distribution network, paving, small medical centers, formal meeting places. According to all, these are signs of progress and well-being and everybody' rights. Moreover, these infrastructures as well as building rehabilitation and scattered secondary housing belonging to outsiders changed the villages appearance and contributed to revitalize them in the 1980s and the 1990s;

(iii) Rebuilding and preservation of collective facilities during the 1990s (e.g. water mill, forge, press, communitarian stable) to protect heritage and attract visitors. Besides no current use or their use being considered obsolete these improvements, supported by the parks and villages authorities with European Union (EU) funding, motivated residents as they are very fond of their origins;

(iv) Stagnation, abandonment and aging, more relevant after 2005. Small medical centers and schools, local and regional services for farmers were disabled and relative recent infrastructures and collective facilities (such as those described above) are closed and abandoned. Building increased but houses are closed as people are now living and working elsewhere. A great majority of the inhabitants are retired and older than 60. All key-informants adverted to a serious lack of children and young and to the fact that common activities may not remain because there is no knowledge transmission, continuity and stability;

(v) Changes in land use and cover. Most of the men and several women emphasized important landscape changing due to a decrease in cattle grazing, quite abandoned meadows and arable lands, the absence of once usual crops (e.g. flax, wheat and hops), the afforestation of individual fields, more diverse homegardens adjacent to new houses, the presence of many cultivated and exotic ornamentals, and fenced fields and plots in opposition to the traditional open and communal spaces;

(vi) More diverse homegardens and new ornamental gardens. Many women highlighted that the number of cultivated species has increased with the introduction of a wide range of greens and ornamental species in the last three decades. These plants or propagation materials have been brought from remote areas by migrants and visitors, exchanged between relatives and neighbors or bought from retailers at the local markets. In order to replicate urban lifestyles, villages' authorities created new areas and gardens where they have introduced exotic herbaceous and woody ornamentals which are, whenever possible, quickly propagated and used in homegardens. Women, who are mainly in charge of these spaces, explained that lifestyle of today is not as demanding as it was in those days, when all agricultural labor was manual and households income was based on agriculture and livestock. So, that leaves more time to care for the garden and flowers. A new concept of villages planning and residential housing also promoted ornamental gardens as dwellings look like villas with courtyard and surrounded by gardens. In former times houses were built side by side, in lines, comprising two floors; animals were kept on the ground floor and the family lived in the first floor. This traditional architecture assured thermal regulation of the houses.

Some changes observed by our informants, such as out-migration effects, abandonment, changing land-use, land cover and landscape are also reported in other European studies [26-30], some conducted in the Iberian Peninsula and Mediterranean area [31-33].

Table 2 resumes how people perceived traditional versus current landscape and summarizes some attributes used by key-informants to refer to both types.

**Table 2:** Perceptions and attributes of traditional versus current landscapes in Montesinho and Douro International Natural Parks, Trás-os-Montes

Traditional landscape	Main sign	Current landscape	Signs of change
Wilderness	Presence of nature	Tameness	Wind turbines, antennas

Continuous	Land cover patterns	Fragmented	Road network

Humanized	Crops and cattle	Abandoned	Scrubland and wildfires risk

Multipurpose	Mosaics of species	Monoculture	Fast growing species

Availability of resources	Wild gathering	Restriction of resource	Hunting regulations

Intensive labor	Crop lands	Absenteeism	Afforestation of crop lands

Landraces	Good performances	Exotic species	Invasive species, diseases

Small settlements	Inconspicuous	Larger villages	Noticeable, modern houses

Communal facilities	Management	Individual facilities	e.g. Many bread ovens

Lack of infrastructures	Rustic and hardy	Infrastructures	Lifestyle improvement

Cultural heritage	Local architecture	Global and homogeneous	Similar public places in several villages

## Discussion

### How natural parks landscapes reflect local knowledge?

The character of traditional landscapes in the study area attests the human presence and LK as they incorporate multipurpose practices and initiatives well adapted to exploit local resources which were fundamental for people survival and welfare.

**Figure 2:**
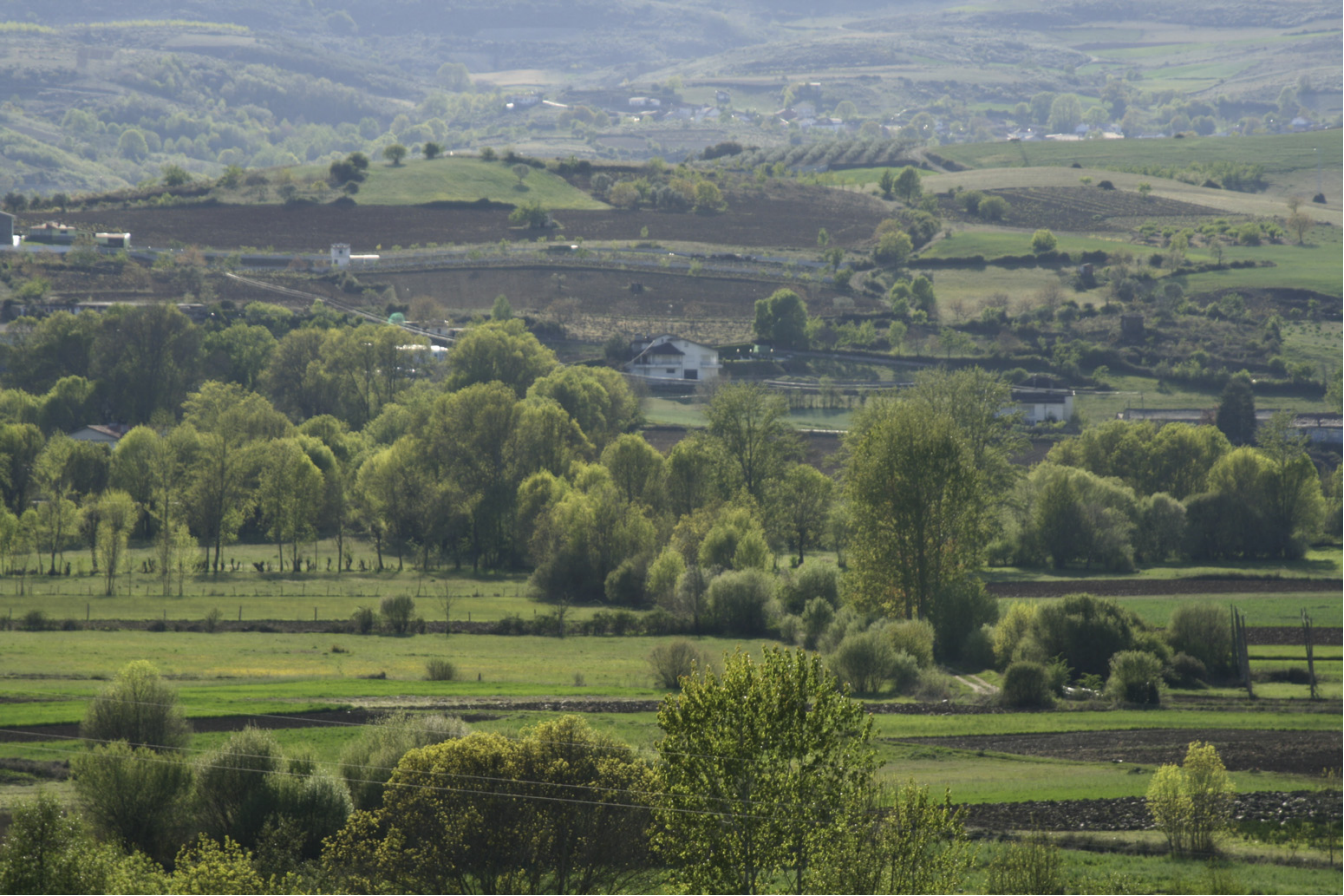
**Traditional landscape of Trás-os-Montes: a mosaic composed of different patches finely linked to each other, mostly highlighted by the seasonal contrasts of natural vegetation and agricultural activities**.

Our respondents have emphasized that people's adaptive management of natural resources, since a long time ago, has built a multifunctional, productive and diverse landscape (Figure 2). Over time, this close relationship between people and their natural and agricultural environment has led to the development of a rich knowledge base on plants, plant-use and related practices.

**Figure 3:**
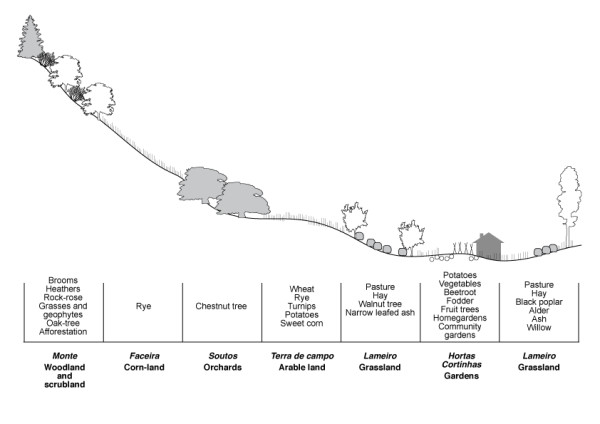
**Land-use system: pattern of species and crops distribution across a longitudinal section, following a decreasing gradient of soil fertility but an increasing gradient in slope and distance to centre**. Adapted from [21].

The long-established land-use system respect a circular configuration (Figure 3) with settlements in the middle, surrounded by homegardens, arable lands, scrubland, woods and crop rotation (rye - more or less long fallow). This pattern follows a decreasing gradient of soil fertility but an increasing gradient in slope and distance to centre; meadows are transversal to these aureoles. Distance to centre is fundamental to the configuration: greater proximity to the village, lower cost of transport (nutrients and products), lower time-consuming, greater return of invested labor and a steady system [34].

Therefore, traditional landscapes are mosaics composed of different patches finely linked to each other, mostly highlighted by the seasonal contrasts of natural vegetation (e.g. perennial or broadleaved forests, woodland and scrubland) and agricultural activities (e.g. fallows, manure, hay or grazed meadows, orchards, gardens). Such landscapes are perceived as images of LK which provide skills and tools to built such a mosaic while maintaining a balance between human activities and nature, and a source of motivation, as they are considered part of the cultural heritage and have embedded intangible values such as dwelling, spiritual and aesthetical values, local tradition, neighborly and inter-generational relations [17,21].

### Why local knowledge is important to plant resources management and conservation?

After the establishment of the protected areas, there was a short period of enthusiastic efforts, policies and regulations that promoted regional economy and revitalized the social and cultural context (e.g. promotion of traditional products, new roads, and health facilities). Reactions to these measures have been generally favorable to the established protected areas and protective rules but their later suspension brought some disillusion to populations and communities [10]. European Community and national policies, along with governmental protective measures required for the establishment of protected areas regimen (e.g the Natura 2000 network), as well as, the lack of financial resources and increased responsibilities with reduced political support have imposed strict production conditions that disabled traditional agricultural activities, weakened motivation and as a consequence interfered with generational transmission processes and the LK system.

Key-informants have pointed out that neither local communities nor resource users have ever been involved in the parks management, although this was initially expected. Their main argument is that national conservation networks and strategies did not take into account regional identity, people background and local believes and habits, i.e. local knowledge. Similar ideas were also found or recommended in other studies around the world [35-37], showing that human-modified landscapes require a range of policy instruments, in addition to the mere implementation of traditional protected areas, that should include an appreciation of local practices and knowledge to maintain the socio-economic system [37].

Moreover, it is perceived that most of the initiatives have never recognized or considered the vital role of human activity in natural protected areas maintenance and the contribution of LK to the current environment. However, several authors [e.g [38,39]] corroborate our informants' perceptions, arguing that humans have lived in the region for millennia and have maintained natural resources sufficient to sustain their livelihoods, from generation to generation, which indicate that they followed practices that promote biodiversity conservation.

We assume there was no global interest in understanding, recognizing and valuing LK which most of the times was/is viewed and connoted old fashion, rustic, too much conservative, relatively limited to subsistence and utilitarian when compared with modern/urban lifestyles.

Therefore, the protected areas of Montesinho and Douro International are suffering a decline as a result of measures that do not promote innovative and participatory approaches based on LK, disclaim cultural heritage and do not provide a recognized/valued lifestyle.

**Figure 4:**
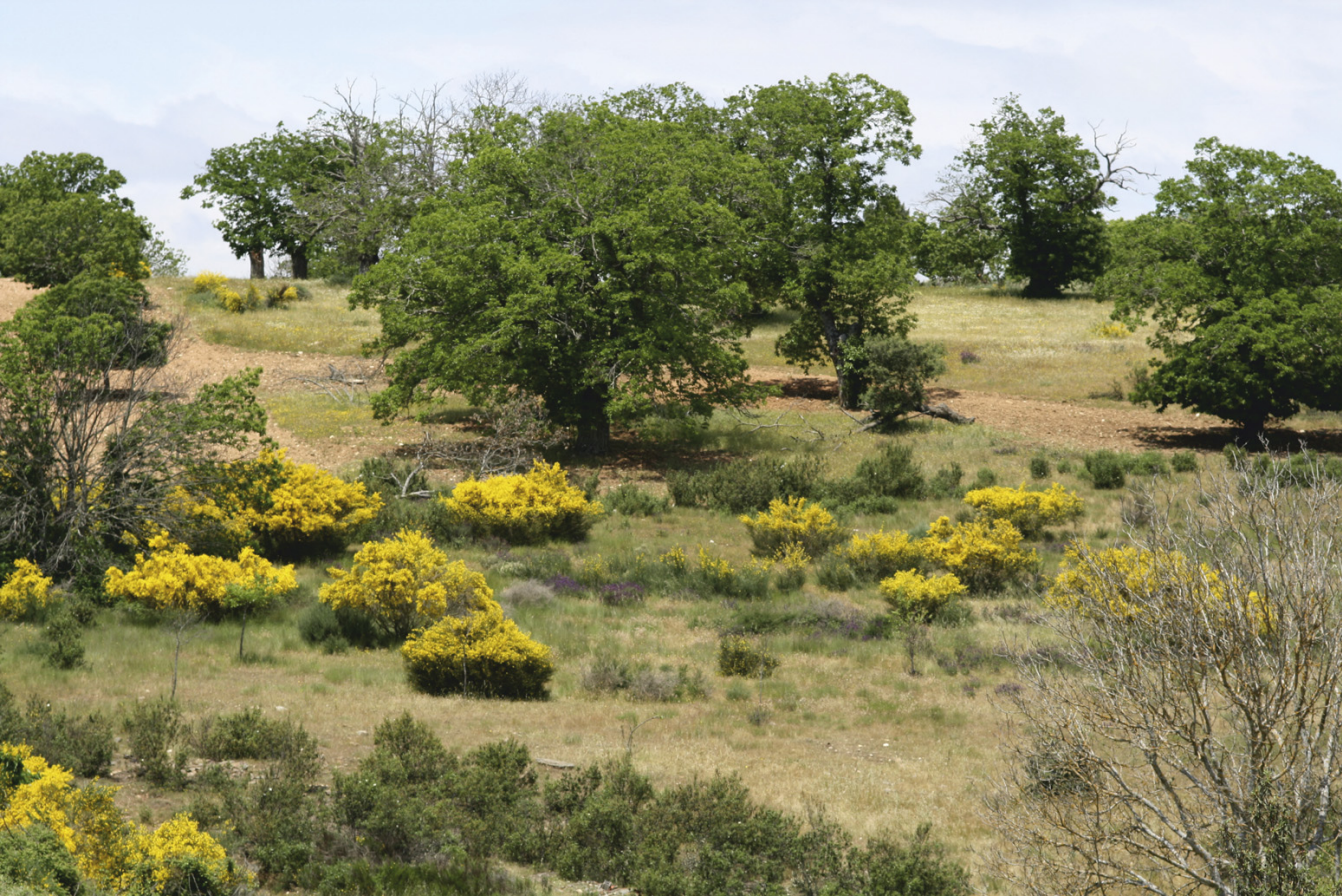
**The signs of land abandonment: lands progressively exhibit a different floristic composition and scrubland is represented by a tallest stratum**.

The harmony between natural environment and the human activities was/is compromised, as well as nature conservation issues and the status of the territories included in the protected areas (Figure 4).

Different issues and examples of LK significance in conservation and management strategies emerged from respondents' comments and may be synthesized as follows:

(i) LK deals with linked practices and skills that may not last alone because they are meaningless without each other.

For instance, grain production and crop rotation are locally coupled with animal husbandry. As people explained, growing wheat or rye usually provided three sub-products: straw for litter and basket weaving, grain for selling in the market and to keep at home, stubbles and fallows for feeding sheep in late summer and after the first autumn rains. Informants were able to remember the enlargement of the areas assigned to rye, wheat and fodder production during the 1950s, as well as the satisfactory performances of local varieties well adapted and the consequent increase of cattle and sheep, which in turn, also concerned the management of the scrublands, meadows and pastures, fallows and stubbles. Cycles of slash-and-burn, cultivation, and scrub were still common in the 1980s. Species from the scrubland were used as fertilizer, litter, pasture, firewood, and some to make charcoal.

A wide variety of wild edibles and medicinal plants used in overlapping contexts are related with the previous mentioned habitats (e.g. crop lands and scrubland). Their prevalence depends on the integrated management of such areas and has serious implications for biodiversity and the status of protected area as well.

As Harrop explained [38], these systems are of such importance that they merit primary support in protected areas. Therefore, there is a need in new instruments or policy documents directly supporting traditional agricultural landscapes [38] and economic incentives can play a central role in policy measures.

(ii) LK is limited by policies that constrain the ability of small farmers to diversify and reduced the mosaic of farming activity.

The Common Agricultural Policy (CAP) aims to provide farmers with a reasonable standard of living and to preserve rural heritage, while guarantying consumers a quality food at fair prices. The specific purposes of increasing agricultural production, providing certainty in food supplies and stabilizing markets have promoted a large expansion in agricultural production often achieved at the expense of non eco-friendly technologies, such as the indiscriminate use of fertilizers and pesticides and the replacement of landraces well adapted. Furthermore, the CAP has usually rewarded those who produce more; larger farms have benefited much more from subsidies than smaller farms.

Obviously, small farmers living inside protected areas find difficult to match these general objectives and agricultural productivity. Their farming systems used to benefit from LK transmitted over centuries and from practices well adapted to local conditions. Such practices were designed to optimize and diversify productivity in the long term rather than maximize it in the short term, avoiding the risk of crop loss. Besides local farmer's ability for innovation, the imposed regulations made impossible to achieve the general goals and to compete with the global market: agriculture was suddenly viewed as an impossible task without competitive advantages because of rising production costs versus low profits and uncertain wages.

Non prevailing agricultural practices promote abandoned arable lands that progressively exhibit a different floristic composition and scrubland represented by a tallest stratum with increased risk of wildfires. Breeders experienced some difficulties to meet municipal ordinances and innovative requirements concerning animal welfare and veterinary care, which entail continued technical assistance. Raising livestock becomes also a difficult task and tends to extinction. As meadows are not cut for hay or grazed the early colonizers are shaded out when woody plants become well-established. Several medicinal plants often gathered in these fields are no more available.

According to our informants these occurrences decrease the intrinsic value of arable and pasture lands and limit the expression of LK because some species, techniques and skills are gradually lost, as well as the social and individual value of their lands.

EU agricultural priorities have been in direct contradiction to long-term goals of the protected areas. These facts bring into question the principles that led to the creation of parks and the ideal of keeping the territories inhabited, particularly while most of the people perceive the parks as something external and limiting.

To find solutions to these problems requires interaction between decision makers and users to explore strategies to improve the economic benefits of multiple land use systems by integrating primary production with recreation, health care and other secondary functions [26]. It is crucial to inventory the options available for improving the cultural identity of specific cultural landscapes, stressing the interrelationships between local cultural (e.g. architecture, art, local traditions) and regional nature-friendly products, based on initiatives from within [26].

(iii) LK public recognition is fundamental to improve communication and cooperation between local communities and protected areas authorities.

Common practices such as hunting, fishing, gathering from the wild, cutting down trees are highly prized and have great cultural significance to the communities of the PNM and PNDI. These activities involve specialized LK that used to be essential for subsistence and turned up important demonstrations of heritage. Nowadays, these practices are highly controlled activities inside both protected areas which is seen as a threat to inhabitants interests.

A lack of communication between stakeholders has been the origin of several misunderstandings concerning land and resources tenure because a local perspective has not been considered and traditional boundaries neither were demarcated nor recognized. In the territories included in the natural parks there are multiple forms of land tenure and complex communitarian rules controlling ownership, access, resources and land use that are overlapped by governmental control through the parks authorities. Property rights are supported by local tenure systems and rules imposed by outsiders have led to rule-breaking and divergences within owners, communities, neighbors, families and visitors.

Particularly mushrooms gathering ruling, the establishment of game reserves and the periods of open season are the topics most contested by key-informants. They think that LK and local expertise would help to solve already existing and potential conflicts. According to some, the park authorities should consider local hunters expertise before making rules.

As these activities have always been important issues for the parks communities, lacking a framework to regulate and optimize their potential in accordance with local and natural values, acknowledging the diversity in communities' values and practices will provide overall enhancement projects, tailored to individual styles and needs.

(iv) LK on natural resources management was/is associated with local perceptions of the environment.

Local ideas of nature have determined distinguishable values of plants and animals and outlined different orientations towards predatory actions. This particular vision legitimizes those predatory activities that could contribute to strike a balance between wild and domesticated resources. According to informants, biological conservation management strategies that follow universal models give more importance to wildlife than to local people and human activities which had the major influence on the shape of the territory as a whole.

For instance, conservation strategies of some species such as the wolf (*Cannis lupus signatus *Cabrera) have implied the restoration of populations of wild prey (e.g. *Cervus elaphus *L., deer and *Capreolus capreolus *L., buck) in order to avoid livestock depredation. However these approaches caused several problems and confrontations. Not only the measures did not avoid wolf predation and economic loss, as the growth of deer population became also out of control and caused substantial damages to crops and homegardens.

People perceived these kinds of strategies without competitive advantages because of uncertain control of wild animal populations versus rising losses and management costs and, apparently, no measurable benefits.

Seemingly, wild prey damages are worse than wolf ones. As the implemented compensation programs are not satisfactory and reimbursements are insufficient and take a long time, some informants think a good solution would be hunting the wild prey, which is only allowed by the parks authorities under particular conditions more favorable to visitors than to residents.

(v) LK may facilitate a multipurpose management of wild and domesticated resources.

Traditional landscapes can provide valuable habitats for many animal species but most of actual plant resources are closely dependent on human management and on socioeconomic and agroecological combined factors for their continued persistence. Many of the traditional agricultural and pastoral practices have been kept up to recent. Local farming systems and agricultural and forestry practices had an extensive character offering suitable habitats and sustaining diverse biological communities.

Moreira and Russo [40], who studied the impact of agricultural abandonment and wildfires on vertebrate diversity in Mediterranean Europe, concluded that loss of animal biodiversity due to agricultural abandonment cannot be compensated by the gain of scrubland and forest species, since, globally, more species are lost than are gained.

Local people would expect that their knowledge and experience along with new concepts and trends in sustainable agriculture and forest management will support innovative and valuable approaches based on regional farm products and on wild plant resources.

Instead, landraces diversity and variability has not been assessed and conserved. Farmers were advised to introduce modern varieties, in order to increase crop yield, and have abandoned their locally adapted ones but with no satisfactory results. Then, afforestation of farmland was regarded as a good alternative to seasonal crops and abandonment because it allows absenteeism, provides income and represents a patrimony for future generations. Arable lands, dry prairies and common lands have been afforested for both timber and fruit production using chestnut, walnut tree, cherry tree and red oak. In wet meadows, fast-growing hybrid poplars were grown on plantations and sold for pulpwood and as inexpensive hardwood timber, used for pallets and cheap plywood. Although it seems a good alternative, several informants commented that there is a risk of plant diseases, such as ink-disease in chestnut. Moreover, seasonal labor for fruit recollection and species management is considered expensive, scarce and difficult to hire.

Once again, local partners had little expectation of success and many of them think that incorporating LK into strategies of conservation (e.g. *in situ *and *ex-situ *conservation strategies for conserving wild and cultivated resources) could have inevitably resulted in sustainable protected areas and would have ensured a reasonable quality of life for their inhabitants. Similar perspectives mentioned in the literature reinforce the view that involving people in management planning and monitoring of parks may increase their support [35-37].

Management strategies of natural parks should guarantee long-term protection and maintenance of biological diversity while providing sustainable natural products and services to prior meet communities' needs.

### Using local knowledge to improve educational and conservation strategies: the Ecomuseum of Terra de Miranda

«More awareness of conservation is an educational issue. Informing people about such issues so that they appreciate the urgency of taking action and the majority support it, making a participatory park a reality» [39].

New insights into the past and contemporary cultural landscapes from these two protected areas which have global ethnoecological importance have provided tangible records of past human use and management of natural resources, bridging practices, representations, expressions, knowledge, skills, as well as instruments, objects, artifacts, dwelling and other domestic and communal spaces.

**Figure 5:**
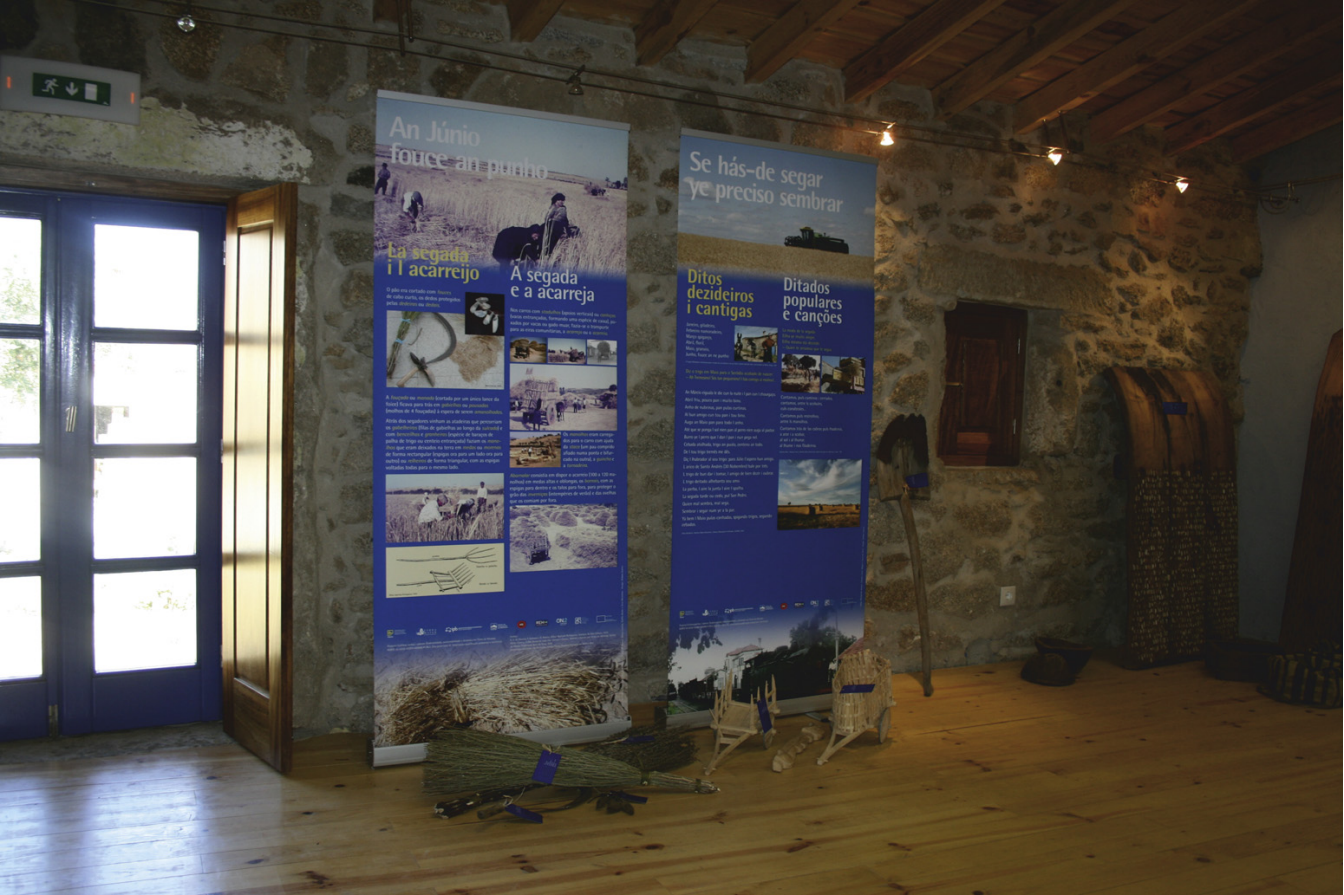
**The Ecomuseum of Terra de Miranda as an example of successful local initiative**.

**Figure 6:**
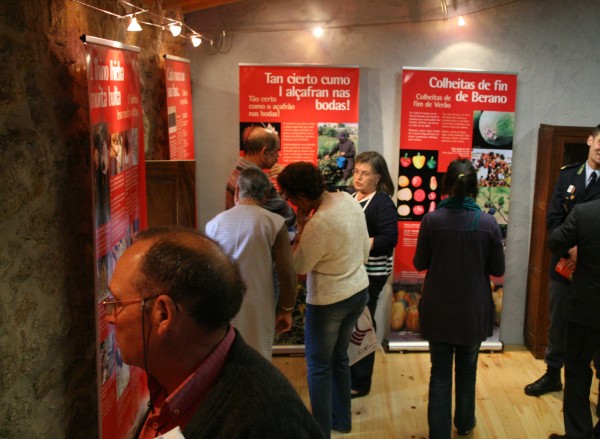
**The Ecomuseum approach seems to be an interesting way of dealing with local knowledge at different levels: communities, authorities and visitors**.

**Figure 7:**
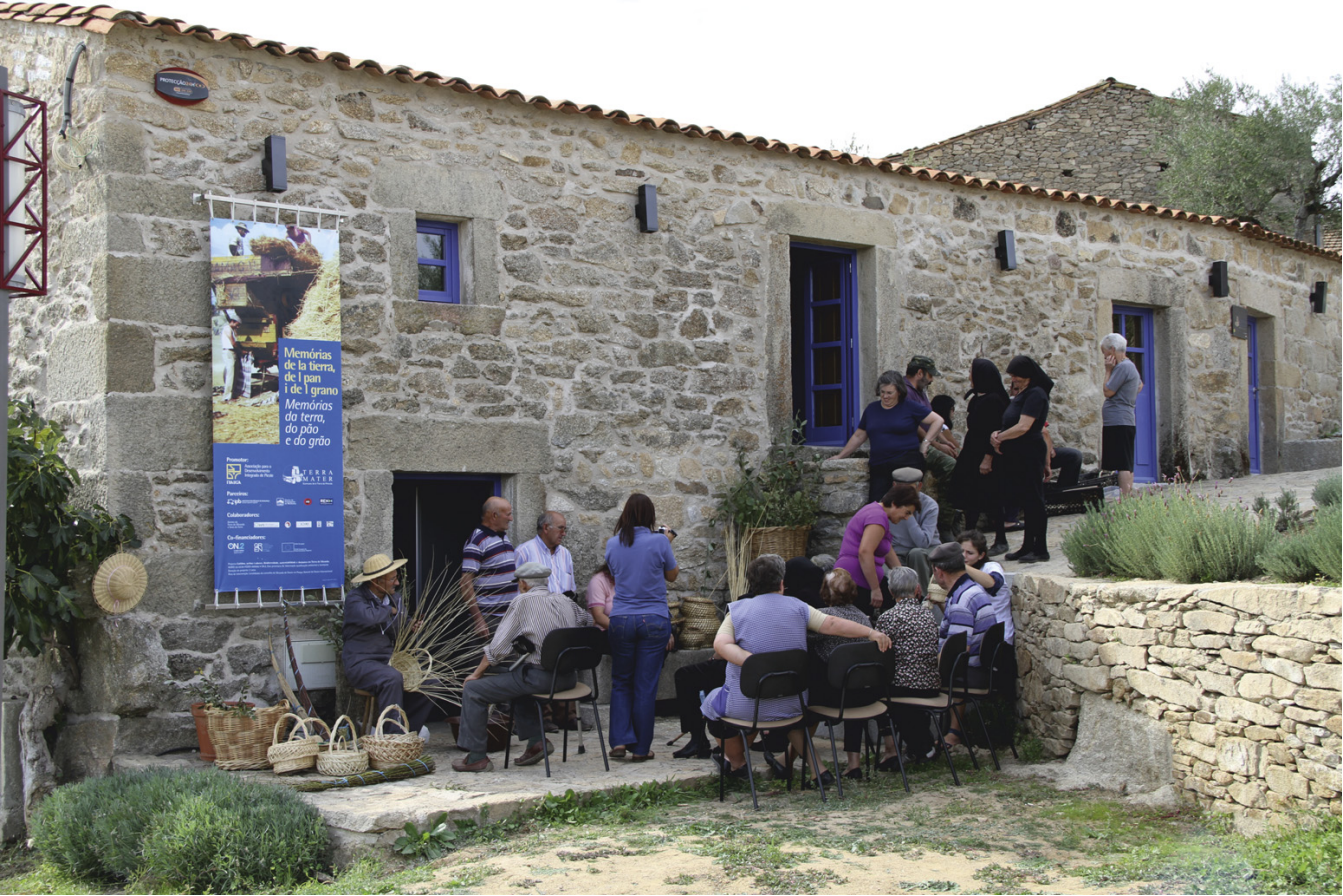
**Local knowledge is an important tool for educational and promotional purposes within natural resources conservation, management and sustainable use**.

The recently created (April 2010) Ecomuseum of Terra de Miranda (Figure 5) in the PNDI territory is an interesting community-based approach to the conservation and management of natural resources, as well as tangible and intangible heritage. The Ecomuseum is a direct result of a local initiative with the PNDI authorities consent. Despite his youth and peripheral location (Miranda do Douro is the most interior northeastern Portuguese region only accessed by an old national road) the Ecomuseum of Terra de Miranda has already developed several educational activities involving seven hundred participants, including local population, children and students, universities and research centers, tourists and public in general (Figures 6 and 7). The Ecomuseum is also trying to promote *in situ *and *ex situ *conservation involving local farmers, homegardeners and the Portuguese Gene Bank.

A multidisciplinary research project aiming to record and document local knowledge and traditional resource management supports the main purposes of different activities and provides multiple solutions to local community involvement. All the organized events have improved local networks, developed training and educational strategies, and made available traditional and new interpretations of cultural heritage. However, an interchangeable role with the PNDI representatives has not been easy to maintain and the inadequacy of some institutional attitudes are the main cause of a certain discomfort and annoyance.

The Ecomuseum approach seems to be an interesting way of dealing with local knowledge at different levels: community, social and cultural, natural environment, institutional. The balance of one year of activities provide valuable insights into perceptions and concepts of local knowledge and of natural resources conservation and management within protected areas established long ago or more recently.

## Conclusion

Cultural landscapes legacy, built over LK, i.e. built over generations of experimentation and observation, provides ideas and opportunities for sustainable and multipurpose use of resources and offers contemporary strategies for preserving cultural and ecological diversity, e.g. intangible and tangible cultural heritage.

Along the interviews, new farming practices, abandonment of farming and husbandry activities, a better mobility and a new concept of residential housing were some mentioned causes for landscape changing which have a direct effect on land-use and plant use and indirectly on LK and generational transmission.

Many of these none prevailing agricultural and management activities were effective in inducing plant-use and LK, e.g. wild plant gathering sites and best periods. People mentioned that "you do not forget what you keep doing"; otherwise most of LK will be lost soon.

It became clear that in the past thirty years, homegardens have become areas of *in situ *and *ex situ *conservation for both nostalgic and pragmatic reasons. Some crops and landraces are no longer cultivated in arable fields and wild species traditionally gathered are threatened by new access roads, wildfires, reforestation activities and land abandonment. Key-informants perceived that young and some middle aged people value some of these changes, which they consider less hard-working and a symbol of modernity allowing a more like urban lifestyle (e.g. weekends and holidays). Others regret actual landscape transformations which they view as a signal of abandonment, waste of resources, and reprehensibly unproductive.

Although legislation on natural parks is very clear about the connection between protecting natural diversity and preserving cultural heritage, people recognize that conservation strategies were set without involving users and communities and incorporating LK. Thus the main objectives of the parks (e.g. conservation, sustainable development, public use and population involvement) have not been fulfilled. Informants suggest that participatory approaches should be implemented in order to adopt measures that will develop more outstanding natural features of protected areas, respecting landscaping, socio-economic and cultural trends and being more favorable to people living in these areas.

LK is often a practical one, based on empirical observation and long experience, and transmitted through oral traditions, but is also a dynamic process that is able to integrate different sources of knowledge. Such knowledge is not merely of academic or historical interest but is fundamental to maintaining cultural identity and useful for providing more realistic evaluations of environment, natural resources and production systems. LK may improve success by involving local people in the planning processes. Pinto [10] who analyzed historical information of the Portuguese protected areas concluded that since local populations living in the protected areas are important for biodiversity conservation, it is recommended that traditional activities continue and that the dialogue between stakeholders is improved. The management of these areas is potentially more efficient if there is a public recognition of their value.

Therefore LK conceptions can be considered important tools for landscape and natural resources conservation, management and sustainable use, also for educational and promotional purposes, as well as for natural protected areas maintenance. To be successful it is absolutely necessary to make people active participants, not simply integrate and validate local knowledge.

## Research projects involved and financial support

Etnobotânica do Parque Natural de Montesinho. PRODEP Program (2000-2005).

Etnobotânica do Nordeste Português: Saberes, plantas e usos. POCI/ANT/59395/2004. FCT, Portuguese Science and Technology Foundation (2004-2008).

Cultibos yerbas i saberes: Biodiversidade, sustentabilidade e dinâmica em Terras de Miranda. NORTE-03-0230-FEDER-000066. ON2, QREN (2009-2011).

## Competing interests

The authors declare that they have no competing interests.

## Images consent statement

All individuals provided consent for publication of all images in the figures.

## Authors' contributions

AMC and AFM conceived the surveys, designed the field work and carried it out. The paper was written by both authors, who read and approved the final manuscript.
